# 

**Published:** 1911-08-26

**Authors:** 


The journal *Paris Médical* has established an information bureau at 19 Rue Hautefeuille, Paris, for the benefit of foreign medical men wishful to obtain information with regard to courses of lectures given at the Faculty of Medicine and in the Paris hospitals. A physician conversant with English, German, Spanish, and Italian attends twice a week to give information to applicants.

### AN UNREGISTERED DENTAL PRACTITIONER.

At Fleetwood Sessions Mr. R. P. Walmsley appeared to answer to a summons for practising as a dentist, he not being a duly registered practitioner. It was stated that on a post-card sent to one of his patients he had used the title "dentist." A fine of 40s. and costs was inflicted.

### BUILDINGS CONTEMPLATED AND IN PROGRESS.

East Sussex.—The Joint Committee of the East Sussex Hospital is inviting architects to submit designs in open competition for a proposed new hospital. Mr. Edwin I. Hall, F.R.I.B.A. is the assessor, and conditions may be had on deposit of 21s. from W. M. Rhodes, Esq., East Sussex Hospital, Hastings.

Poplar and Stepney.—The Managers are prepared to receive tenders for painting and cleaning at the Blackwall branch asylum. Firms tendering will be required to declare that they pay the rates of wages and observe the hours of labour that are generally recognised by the Trades Unions and considered fair in the respective trades.

Reading.—The Reading Town Council have decided to provide additional hospital accommodation for the treatment of children in the early stages of tuberculosis and for this purpose are negotiating with landowners.

Rochdale.—The Board of the Rochdale Infirmary have decided to proceed with a portion of a large extension scheme to cost about £16,000. It will include an operating theatre, staff accommodation, ophthalmic rooms, modernised in-patients' department, and an additional twenty-four beds. The assessor appointed for the limited competition in which provision is to be made for forty-eight beds at a cost of not more than £17,000, is Mr. Alexander Graham, F.R.I.B.A., of London.

Rottingdean.—Mr. J. W. Hawker, of Brighton, is architect for the erection of receiving-homes and infirmary at Rottingdean. Seven tenders were received, the lowest, £7,745, is provisionally accepted. The highest amounted to £8,732.

Springfield.—The Fife and Kinross Lunacy Board propose to add a hundred additional beds to the Springfield Asylum.

Staffordshire.—For additions to the Albert Ward at the North Staffordshire Infirmary Messrs. Scrivener and Sons, of Hanley, are architects. Five tenders have been received for the work—the highest, £1,375; the lowest, £1,178.

Thornton, Fifeshire.—Messrs. Gillespie and Scott, the well-known architects practising in St. Andrews, have submitted a scheme for a new hospital wing at the Thornton Poorhouse giving accommodation for sixty-one inmates, at an estimated cost of £7,200, and also a suggestion for more modern kitchen and laundry buildings at an approximate cost of £2,800.

West Derby (Lancs.).—Additions to the infirmary in Mill Road are proposed, and the estimated cost is £4,050. The work is being carried out for the West Derby Board of Guardians.

West Ham.—The Council invite tenders for new dormitory buildings at the Convalescent Home, Harold Wood, Essex. Sureties are required, and there is a fair-wages clause in the contract form.

Winwick (Warwick).—An extension of the Nurses' Home is proposed. Specification and quantities may be obtained from the Clerk and Steward, Winwick Asylum, on deposit of half a guinea.

### THE HOSPITAL WORLD.

### BOOKS RECEIVED.

James and Sons.

"Physiology of the Central Nervous System." By N. J. Vazifdar.

John Wright and Sons, Ltd.

"Lateral Curvature of the Spine." By J. T. Kellett Smith.

Williams and Norgate.

"Health and Disease." By W. L. Mackenzie.

Scientific Press, Ltd.

"Medical Homes for Private Patients." Edited by R. Pritchard Birnie.

John Bale, Son and Danielsson, Ltd.

"Theory and Practice of Thyroid Therapy." By Herbert Ewan Waller.

### THE WEEK'S NOVELTIES.

### A NEW BED FOR THE TROPICS.

Among the latest types of hospital bedsteads are the patent "Wheelingout" pattern which Messrs. J. Nesbit-Evans and Co. have recently brought out. This firm is also responsible for a patented bed-inclining stand, which is peculiar in that it can be applied either to the head or the foot of the bedstead. An illustration of it can be seen in our advertisement columns. Nesbit-Evans and Co. have also put on the market a bed designed for Indian hospitals, with a mosquito-curtain arrangement suitable for the tropics. Aseptic furniture is aimed at in the white-enamelled lockers and bedside tables that can be obtained from the company's Birmingham works in Floodgate Street.

### GIFTS.

Miss Frances Jane Hazard, of Harrogate, has bequeathed £50 to the Harrogate Infirmary.

Mr. Alexander Henry Clarke, of Earl's Court, S.W., solicitor, has left £500 each to Middlesex Hospital, King's College Hospital, and the Cancer Hospital, Brompton.

Mrs. Susan Aldam Milner, of Sheffield, left £50 each to the Sheffield Public Hospital and Dispensary, the Sheffield General Infirmary, and to the Jessop Hospital for Women.

Mr. John Bussey, of Carnarvon, formerly of Bilton, Staffs, grocer, has left £300 to the Wolverhampton and Staffordshire General Hospital, and £100 to the Wolverhampton Eye Infirmary.

Miss Ellen Burmester, of Hyde Park, W., has left £500 each to the Royal Hospital for Incurables, Putney, the Royal Free Hospital, Gray's Inn Road, W.C., the London Ophthalmic Hospital, City Road, the British Home for Incurables, Streatham, and £250 each to the Hospital for Sick Children, Great Ormond Street, W.C., and to the Marylebone Home for Incurables.

### APPOINTMENTS VACANT.

Full particulars of these vacancies can be obtained on reference to our advertisement columns :

Bradford Royal Infirmary.—House Physician and Two House Surgeons.

Cameron Hospital, West Hartlepool.—House Surgeon.

Devonshire Hospital, Buxton, Derbyshire.—Assistant House Surgeon.

Liverpool Royal Southern Hospital.—House Surgeons and House Physicians.

Manchester Children's Hospital.—Visiting Surgeon and Male Resident Medical Officer.

Queen's Hospital for Children, Hackney Road.—House Surgeon.

Royal Albert Hospital, Devonport.—Assistant House Surgeon.

Royal South Hants and Southampton Hospital.—House Physician.

Sunderland Royal Infirmary.—House Physician.

### TO HOSPITAL SECRETARIES AND OTHERS.

We invite contributions from any of our readers, and shall be glad to pay, according to length, for items of news for the institutional section (including appointments, resignations, gifts, etc.) which we may publish. All payments for manuscripts used are made as early as possible after the beginning of each quarter.

